# Evidence for immortality and autonomy in animal cancer models is often not provided, which causes confusion on key issues of cancer biology

**DOI:** 10.7150/jca.41324

**Published:** 2020-03-04

**Authors:** Xixi Dou, Pingzhen Tong, Hai Huang, Lucas Zellmer, Yan He, Qingwen Jia, Daizhou Zhang, Jiang Peng, Chenguang Wang, Ningzhi Xu, Dezhong Joshua Liao

**Affiliations:** 1 Shandong Provincial Key Laboratory of Transmucosal and Transdermal Drug Delivery, Shandong Freda Pharmaceutical Group Co., Ltd., Jinan 250101, Shandong Province, P.R. China.; 2 Department of Pathology, The Second Hospital of Guizhou University of Traditional Chinese Medicine, Guiyang 550001, Guizhou Province, P.R. China.; 3 Center for Clinical Laboratories, The Affiliated Hospital of Guizhou Medical University, Guiyang 550004, Guizhou Province, P.R. China.; 4 Masonic Cancer Center, University of Minnesota, 435 E. River Road, Minneapolis, MN 55455, USA.; 5 Key Lab of Endemic and Ethnic Diseases of The Ministry of Education of China in Guizhou Medical University, Guiyang, Guizhou Province 550004, P. R. China.; 6 Department of Orthopaedics, Shandong Provincial Hospital Affiliated to Shandong University, Jinan 250021, Shandong Province, P.R. China.; 7 Tianjin LIPOGEN Gene Technology Ltd., #238 Baidi Road, Nankai District, Tianjin 300192, P.R. China.; 8 Laboratory of Cell and Molecular Biology & State Key Laboratory of Molecular Oncology, National Cancer Center/Cancer Hospital, Chinese Academy of Medical Sciences, Beijing 100021, P.R. China.

**Keywords:** Transgenic, cancer, carcinogenesis, immortality, autonomy, cancer stem cells, senescence.

## Abstract

Modern research into carcinogenesis has undergone three phases. Surgeons and pathologists started the first phase roughly 250 years ago, establishing morphological traits of tumors for pathologic diagnosis, and setting immortality and autonomy as indispensable criteria for neoplasms. A century ago, medical doctors, biologists and chemists started to enhance “experimental cancer research” by establishing many animal models of chemical-induced carcinogenesis for studies of cellular mechanisms. In this second phase, the two-hit theory and stepwise carcinogenesis of “initiation-promotion” or “initiation-promotion-progression” were established, with an illustrious finding that outgrowths induced in animals depend on the inducers, and thus are not authentically neoplastic, until late stages. The last 40 years are the third incarnation, molecular biologists have gradually dominated the carcinogenesis research fraternity and have established numerous genetically-modified animal models of carcinogenesis. However, evidence has not been provided for immortality and autonomy of the lesions from most of these models. Probably, many lesions had already been collected from animals for analyses of molecular mechanisms of “cancer” before the lesions became autonomous. We herein review the monumental work of many predecessors to reinforce that evidence for immortality and autonomy is essential for confirming a neoplastic nature. We extrapolate that immortality and autonomy are established early during sporadic human carcinogenesis, unlike the late establishment in most animal models. It is imperative to resume many forerunners' work by determining the genetic bases for initiation, promotion and progression, the genetic bases for immortality and autonomy, and which animal models are, in fact, good for identifying such genetic bases.

## Introduction

Cancer research has been going on for 2,700 years 1, but in our opinion systematic studies of cancer had not been expedited until the 1760s when several historical events occurred that greatly accelerated the research: First, in 1761 John Hill published his observation of cancers in the nasal cavity of snuff users, which is the first report on a connection between chemicals and human cancer 2. Second, in 1775 Pott reported his “chirurgical” observation of cancers in several body sites and identified cancer in the scrotal skin of British chimney sweeps 3. Because chimney sweeps in other European countries did not have this cancer, it was suspected that British chimney sweeps, unlike those in other countries, did not bathe as a matter of honor, which allowed carcinogenic hydrocarbons from soot to be retained on the scrotal skin. Ensuing requirement of bathing at least once a week, recommended by Hill, significantly prevented this cancer occurrence. This is the first documented success in cancer prevention, as described by Sell 4. Third, according to Triolo's comprehensive review 5, the French Royal Academy of Surgery in 1772 and the Lyon Academy of Science in 1733 offered prizes for original essays on the question of “what is cancer”, which drove a national-wide search for the nature and definition of cancer. Fourth, the latter prize mentioned above was awarded to the surgeon-chemist Bernard Peyrilhe for his work on the inoculation of dogs with human-derived cancer fluid, which was published in 1774 as the first documented animal experiment on cancer in the literature 5, to our knowledge.

One of the results from the last 250 or so years of extensive research on cancer is the so enormous size of the literature that often becomes a tribulation for researchers. Actually, even in 1955, Alexander Haddow (1907-1976) had pointed this out, writing that “the mere abundance of the data …presents a growing problem, towards which there are two extreme types of reaction: first, that of the happy researcher who is content to ignore the original literature, and to rely upon others for his information; and secondly, the reaction of those whom the literature totally enslaves” 6. Few of today's researchers peruse the ancient literature due to their many tiers of stress, such as dwindling funding and increasing difficulty in obtaining tenured faculty positions, besides the colossal volume of the literature to read. As a repercussion, cancer research has manifested a discontinuous growth, just like cancer itself that is a discontinuous growth from its normal parental cell, meaning that few of today's cancer students know and address the questions raised by their predecessors.

We write this perspective article to review some seminal findings by different trailblazers in “carcinogenesis research”, which is herein defined as the study 1) on the procedures that convert a normal cell to a malignant one and then to more malignant states, and 2) on the mechanisms underlying these steps. Cancer's clinical quarter will not be touched upon to avoid digression. The idiom of “tumorigenesis”, which is of broader scope as it also covers the formation of benign tumors, is used sometimes, partly because many animal models produce both benign and malignant lesions.

In our opinion, modern research on tumorigenesis has undergone three phases. The first one began in the late 18^th^ century and went through the entire 19^th^ century 5, 7, 8. The cancer research fraternity in this phase was dominated by surgeons, pathologists, and anatomists. They established autopsy and biopsy as the routine pathology practice, which led to the establishment of the morphological traits of neoplasms 5, 9 and some theoretical achievements, such as the supposition by Virchow (1821-1902) that cancer resulted from chronic irritation 10-13, mainly inflammation 7, 8, 14. The second phase had its inception roughly at the beginning of the 20^th^ century and was coined as an epoch of “experimental cancer research” by prominent cancer pathologists James Ewing (1866-1943), according to Cardiff and Kenney 15, and Harold Leroy Stewart (1908-1998) 9. In this incarnation, medical doctors, biologists, and chemists established and characterized many animal models of chemical-induced tumorigenesis 16. Using these models, they established the two-hit theory 17-21, mutation theory 22-24, clonal evolution theory 25-28, as well as the multi-stage 29-32, i.e. initiation-promotion 33-38 or initiation-promotion-progression 39-44, models of carcinogenesis. Animal models of irradiation-induced carcinogenesis emerged during this period as well 45.

Starting about 40 years ago, molecular biologists, many lacking strict training and clinical experience in surgical pathology or oncology, have gradually replaced medical doctors and traditional biologists to now dominate the fraternity of carcinogenesis research 46, 47, thus moving “experimental cancer research” into a new phase. In this latest incarnation, molecular biologists have established numerous genetically manipulated animal models of carcinogenesis and *in vitro* systems of neoplastic transformation of normal cells which have led us to deeper mechanisms of how genes regulate behaviors of normal and neoplastic cells. We now enjoy enormous amounts of information and great details on molecular signaling pathways for almost all physiological functions and pathological alterations in the human body. However, few of the genetic animal models established so far address the traditional multiple stages of “initiation-promotion-progression” 41, leaving those mavens who are familiar with their predecessors' work to wonder how to couple the stepwise biological changes observed previously with the molecular alterations seen in these genetic models. Moreover, few of the publications reporting these genetic models provide material evidence for immortality and autonomy of the resulting lesions. To warrant this statement, we encourage readers to search published reports of these genetic models for “immortal”, “autonomous”, or similar keywords, to see how many of them describe these properties of the resulting lesions. By reviewing the work of many forerunners, most being preeminent cancer pathologists, we attempt in this essay to reinforce immortality and autonomy as the cardinal, yet long-neglected, criteria to qualify outgrowths as neoplasms.

### Many chemical-induced tumors in animals remain dependent on that chemical until late stages

To our knowledge, the first experimentally induced tumors in animals were reported, in the German literature, by Ledoux-Lebard in 1885 48, who, according to Triolo 7, observed epithelioma in the lungs of the rabbits injected with a mixture of sweet almond oil and croton oil. In 1900, Brosch induced atypical epithelial growths in the crushed skin of a guinea pig with applications of a xylol-paraffin solution 49. As described by Davis 50, 51 and Vasiliev 52, in 1906, Fischer showed that subcutaneous injections of Scarlet Red into the ears of rabbits induced papilloma, which regressed upon discontinuation of the injections but reappeared with further injections 53. According to Davis 50, 51, these phenomena were confirmed by Helmholz in 1907 and by Werner in 1908. Between 1914 and 1924, Katsusaburo Yamagiwa (1863-1930), after he left Virchow and returned to Japan 54, 55, induced papilloma and papillocarcinoma in rabbits' ears by painting the ears with coal tar; metastases were seen in lymph nodes in some cases. However, the tumors regressed upon cessation of the tar-painting but recurred quickly if the painting resumed 56, 57. Yamagiwa thus concluded that “carcinomas do not develop as carcinomas from the beginning, and do not always continue as carcinomas” 57. This “do not always continue” is the first statement in the literature, to our knowledge, stating that induced cancer can disappear spontaneously. During 1930s and 1940s, Peyton Rous (1879-1970), a Nobel laureate, confirmed the regression of the lesions upon tar discontinuation and their quick reappearance upon tar repainting 38, 58, 59. Actually, according to Rous, Des Ligneris had already confirmed in 1930 that,“…a second period of tarring brings out warts sooner than the first” 58. Realizing that the reversible lesions could not be authentic neoplasms, Rous described them as warts, which are hyperplastic lesions, and wrote in 1940 that, “…it will be seen that the tar warts of rabbits are tumors by all of the standard criteria except two. They have no capacity for independent growth like that exhibited by most (but not all) classical tumors; and the changes in their cells may conceivably be reversible since they often become smaller and vanish” 58. The two unmet criteria in Rous' observations, i.e. “no capacity for independent growth” and being “reversible”, are later referred to as “autonomy” and “immortality” in the literature. Rous further wrote that, “…in the current definition of a tumor no allowance is made for neoplasms which depend upon favoring factors for existence, and it cannot be used to rule them out” 58. Here, the lesion inducer is dubbed as “favoring factors”.

Chronic treatment of rats with 7,12-diemthylbenz(a)anthracene (DMBA) can induce mammary tumors, but sustenance of the tumors requires continuation of DMBA 60-63. Continuous feeding of rats with 3-methylcholanthrene could induce palpable mammary tumors as early as the 20th day from the start of the feeding 64. Painting the skin of C57 brown mice with 3-methylcholanthrene could also induce palpable tumors as early as the 31st day 65, but 15 of the 22 induced skin tumors regressed completely upon cessation of the inducer and only three of the persisting 15 evolved to histological malignancies 65. Similarly, a large number of papilloma could be induced by painting the skin of albino mice with 3:4-benzyprene, but the tumors actually sloughed off and only a few progressed to carcinomas 66. After having studied successive stages of carcinogenesis 67-70, Rusch wrote in 1950 that carcinogenesis generally consisted of “induction, reversibility and progression” 71, which clearly points out that lesions can be immortal and autonomous only at a late stage. The typical inducer-dependency until a late stage can be exemplified by the skin carcinogenesis model presented by Berenblum in 1947 34, which, in Haddow's words, “proceeds from the normal epithelium first to an early non-specific hyperplasia, second to a specific pre-neoplastic hyperplasia, and then to the emergence of papillomata, and how later stages can be recognized in the progressive growth of such papillomata, their conversion into carcinoma, and the uncontrolled growth of the latter…This general sequence takes place equally well whether exposure to the carcinogen is continued or not” 72.

### Hormone-induced tumors in animals are inducer-dependent until late stages

As comprehensively reviewed by Cardiff and N Kenney 15, breast cancer has been known to be regulated by female hormones since 1896 when Beatson reported the regression and recurrence of a breast lump in a 33-year-old woman following removal of her ovaries 73; other similar cases were also reported in the following years 74, 75. Lathrop and Loeb also reported similar findings in spayed mice in 1916 76. Chronic treatment of rats and mice with estrogens can induce cancers in the bladder and mammary glands and benign tumors in the pituitary and testes 77-92, and can also induce uterine tumors 93. The ACI strain (August strain crossed with Copenhagen strain, also called AxC) of rats may be more susceptible than other strains to the induction of the mammary and pituitary tumors 78, 94, but we once found that about one-fourth of the females lacked one side of the uterus and ovary (DJ Liao's unpublished data), suggesting that the ACI strain may bear a recessive mutation in a relevant but not yet identified gene. Treatment of mice with estrogen, or with both estrogen and androgen, can induce benign and malignant tumors in the cervix and vagina; these malignant tumors are transplantable to other mice treated with the hormones 95-101. Administration of androgens to rats can induce prostate 102-106 and uterine 107, 108 cancers. Concomitant treatment of rats with estrogen and androgen can induce mammary and prostate cancers much more quickly than treatment with androgen or estrogen alone 102-106, 109-114 and can induce uterine leiomyomas as well 115. Administration of estrogen to hamsters can induce malignant renal tumors with abdominal metastases 116-121, while administration of both estrogen and androgen to hamsters can induce malignant tumors in the kidneys and induce benign and malignant tumors in the uterus, in the skin, and in the epididymal tail and adjacent ductus deferens 102, 122, 123. Moreover, gonadal and gonadotrophic hormones have also been shown to possess the ability to induce endocrine cell tumors in ovaries or testes 90, 124-127. Transplantation of the ovary into the spleen can cause neoplasia of the ovary as well, because it eliminates the feedback control regulating hormonal synthesis in the ovary and provides the ovary with unrequited stimulus of pituitary hormones 128.

Estrogen-induced mammary cancer, as well as pituitary and testicular tumors, have been known since the 1930s to regress partially or completely upon withdrawal of the hormone, and the tumors can sustain themselves without estrogen treatment only at very late stages 77, 87, 100, 129-146 (and DJ Liao's personal experience). Initially, estrogen-induced pituitary tumors can be transplanted only to animals treated with estrogens, but not to the untreated animals, evincing their dependency on an excessive amount of estrogen 77. However, they can eventually evolve to estrogen-independency 147, 148. The Nobel laureate Charles B. Huggins (1901-1997) had shown in both animal studies and human clinics that castration or treatment with estrogens could cause regression of prostate cancer at certain stages, signifying that this cancer is hormone-dependent until a late stage 149-151. In the words of Jacob Furth (1896-1979), a renowned pathologist 152-154, “this (prostate) tumor is an example of a growth in man with a spectrum ranging from conditioned to highly autonomous type. The cases of Huggins that were controlled by castration (that is, removal of sources of androgens) may be regarded as dependent; those which partially or temporarily regressed after castration or estrogen treatment, as partially dependent; those not influenced by such therapy, as autonomous” 155. Estrogen-induced renal tumors in hamsters, including their abdominal metastases, will regress upon cessation of the estrogen treatment unless the tumors are at very advanced stages 156-159 (and DJ Liao's empirical knowledge). Initially, these renal tumors are transplantable only to those hamsters that are treated with estrogen, connoting that the tumors still depend on an excessive amount of estrogen, but autonomy can eventually be achieved by manipulation of the estrogen in the recipient animals 119, 159, 160. The hormone dependency seen in all of these studies is the rationale behind the anti-hormone treatments of hormone-dependent cancers 161-163.

Treatment of mice with iodine-131 (I-131) or other anti-thyroid drugs can induce pituitary tumors that secrete thyroid stimulating hormone (TSH) 77, 164-168 because the drugs damage the thyroid and thus decrease the levels of thyroid hormones. This, in turn, stimulates proliferation of TSH secretory cells in the pituitary 169-173. By the same principle, partial thyroidectomy of rats and mice can cause pituitary adenomas as well 174-179. The tumors can be transplanted 180; initially only to those mice treated with thiouracil or other goitrogenic compounds that induce TSH and then gradually to normal mice 181, which again shows the trajectory of “initial dependence and then autonomy”.

Thyroid neoplasms can be induced in mice by treatment with thiouracil or other goitrogenic compounds 174, 177, 178, 182-192 or with I-131 171, 193-196 as a sequel of a high amount of TSH secreted from the pituitary. These thyroid tumors are TSH-dependent but often metastasize to lymph nodes 155 and the lungs 155, 174, 185, although the tumor cells can be converted to TSH-independence via continuous subpassage in culture 155, 174, 177, 178, 183. The hormone dependency of thyroid tumors, including adenocarcinomas, also occurs in a Zebrafish colony as feeding the fish with salt that contains iodine causes regression of the tumors 197. In 1953, Furth wrote that “conditions can be created whereby uncontrolled proliferation of one cell type is obtained, resulting in a tumor-like growth. Manipulations attaining this need not involve any intrinsic alteration in cells causing them to behave as cancer cells. Whether or not such tumors and the similar human metastasizing thyroid adenomas are considered neoplastic depends on the definition of a neoplasm. In our terminology such thyroid tumors are conditioned neoplasms. In the course of subpassage in thiouracil-treated mice the dependent growths give rise to autonomous growths which possess individual features of their own and can be grafted on normal mice. Thyroid adenomas induced by TSH-secreting pituitary tumors are indistinguishable from those induced by thiouracil” 155. Here, Furth used “tumor-like growth”, “behave as cancer cells”, and “conditional neoplasms” to express his reservation in considering the induced pituitary and thyroid tumors, even the spontaneous human thyroid tumors, as authentic; despite their ability to metastasize. In his punditry, “dependent tumors are those in which apparently normal cells proliferate in an altered host; autonomous tumors are those in which permanently altered cells proliferate in normal hosts” 155, although, based on our training in human pathology, we opine that dependent “tumor cells” are not normal but are hyperplastic.

### Some tumors from genetically manipulated animals are inducer-dependent as well

The c-myc gene or a k-ras mutant can induce malignant tumors in many lines of transgenic mice, as we have shown or reviewed before 139,198-205. However, many of the tumors have been shown to regress upon turning off the transgene and can be sustained without the expression of the transgene only at advanced stages, although, once they have regressed, they can be quickly re-induced by reactivation of the transgene 205-223. Xmrk, c-myc, mutant k-ras, or SV40 large T oncogene can also induce liver cancer in transgenic Zebrafish, and again, the tumors will regress after inactivation of the transgene 224-230. Conversely, inactivation of the tumor suppressor gene p53 via conditional knockout can beget tumor formation, but reactivation of the p53 leads to regression of the tumors 228, 229, 231-235. This phenomenon of “regression upon inducer withdrawal and quick repopulation upon reintroduction of the inducer” is a full reflection of the same phenomenon seen in the chemical- or hormone-induced carcinogenesis described above, and has become a rationale for targeting therapy in cancer 236-238. Our contemporaries in the third phase of carcinogenesis research consider “regression upon inducer withdrawal” as “oncogene addiction” and “tumor dormancy” as the reason for “the tumor repopulation upon reintroduction of the inducer” 206, 210, 213, 216-218, 236-239, but, peculiarly, without mentioning the same phenomenon observed by our predecessors.

### Spontaneous regression of human neoplasms occurs but is rare

In humans, spontaneous regression or remission of a neoplasm is extremely rare, but it is recurrently shown in case reports 240-255 with a frequency varying between 1/60,000 and 1/140,000 cases 242, 256-258. Malignant melanoma may have the highest rate of spontaneous regression 259-265. Ever since its first case reported in 1866, as reviewed by Kalialis 266, it has been reported that 10-50% of cutaneous malignant melanoma cases show partial or complete regression without treatment 267, 268, including 0.23% of the metastatic cases 267. High rates of spontaneous regression have also been reported for indolent histologic subtypes of non-Hodgkin's lymphoma, varying between 10% and 20% in selected series, as reviewed by Drobyski and Qazi 269. Pediatric neuroblastoma is another malignancy with a high frequency of spontaneous regression, especially those cases categorized into stage IV-S 270,271. Other types of cancer often showing spontaneous regression include renal cell carcinoma, choriocarcinoma, lymphoid malignancies, etc. 240-242, 272-275.

### Some sporadic tumors in animals and plants also regress spontaneously in a seasonal manner

Spontaneous regression also occurs in animal tumors, such as in mice 276. Mention should be made of tumors in some species of fish and amphibians that often regress spontaneously in a seasonal or temperature-sensitive manner 277-285. The ambient temperatures in some seasons may be hostile for the tumor-inciting micropathogens to grow, and thus fewer tumors occur, but it remains obscure why overt tumors in these cold-blooded creatures disappear in these seasons. The fact that the fish or amphibians themselves live well while the tumors are sloughed off suggests that the tumors require a different microenvironment to sustain their autonomous lives. Similarly, it has also been known for almost a century that some plants will not develop tumors at some hot ambient temperatures 286, albeit both the plants and the tumor-inciting micropathogens can grow happily at those temperatures 286-290. Whether overt tumors in these plants will regress at a hostile temperature remains unknown.

### Immortality and autonomy had already become indispensable criteria for neoplasms a century ago

The studies described above on chemical- or hormone-induced outgrowths are among the earliest ones that point out the problem of “inducer-dependency” and set immortality and autonomy as criteria for neoplasms. Actually, as reviewed by Triolo in 1965 5, research on human cancers in the 19^th^ century had already led researchers, mainly surgeons and pathologists, to a theory that, “cancer cells are autonomous, endow themselves with the power of an independent existence, and divert their entire resources into an unlimited capacity for growth.” This theory finally entered into a rudimentary form and was given as a formal introduction of cancer by J. George Adami in 1901 291 and, according to Rous 58, as a cancer definition in some German pathology textbooks published in the 1910s.

Furth 155 and Ewing 292 considered that all tumors should be in some form of autonomy. Haddow wrote in the 1947 that, “…we now know that, while constitutional and genetic factors can greatly influence susceptibility to cancer, and many even determine the site of its spontaneous occurrence, the disease is one of the individual cells as a separate organism and with no relation to the needs of the body as a whole. It is this which gives cancer its unique position in pathology, accounts for its intractable nature, and explains its growth, in Paget's words 'irrespective of the maintenance of the rest of the body, discordant from its normal type, and with no seeming purpose' (Paget, 1853)” 72. The quoted words of Paget had already, in 1853, pointed out the tumors' autonomous nature. Indeed, according to Haddow 72 and Knauss et al 293, a cancer has long been regarded as a new race or new strain of organism, which is another way of describing autonomy dating back to 1897 by David Hansemann, 1903 by G. Hauser (Beitr. Path. Anat., 1903; 33, 1), and 1926 by Menetrier. Many other former pundits also described carcinogenesis as an atavistic procedure, further pointing out that the resulting “new race of organism” is evolutionarily-lower than its host animal 294-301.

Immortality betokens that a tumor can survive as a “newly developed independent organism” 58 that parasitizes the host 294, 295 and forever maintains its life by continuous replication of its cells 296, 302, 303. As adduced by Paget in 1889 304, “as Langenbeck says, every single cancer cell must be regarded as an organism, alive and capable of development.” Harry Greene (1904-1969), a preeminent surgical pathologist at Yale University, elaborated on the autonomy by writing in 1951 that, “…the definition of a tumor as an autonomous growth has enjoyed persistent popularity in textbooks of pathology. In such definitions the adjective 'autonomous' is employed to express the idea of independence with respect to two different particulars. One of these relates to freedom from the laws restraining and coordinating normal tissue growth, and the other concerns release from the necessity of a continued stimulus” 305. According to Furth's translation 155, in an article written in German from 1951, Bungeler considered that a dependence seen in a large variety of human outgrowths indicates that the outgrowths are not true tumors and, more critically, there is no transition between the dependent and autonomous outgrowths. This “no transition” means that whether an outgrowth is autonomous or not is a black-and-white demarcation between neoplasms and non-neoplasms. Describing human cancer's properties, Emmanuel Farber (1918-2014), a superlative cancer pathologist, also accentuated autonomy as a cornerstone of cancer biology 306. Notwithstanding, it still needs to be pointed out that autonomy of tumor cells may be achieved via non-autonomous mechanisms, e.g. various interactions with other cell types 307-309.

### Mutation and inauthenticity may explain some cases of spontaneous regression

Since malignant tumors keep randomly mutating, theoretically some mutations may be good ones that direct the cells to differentiation or facilitate clearance of tumor cells by immune cells, such as mutation of the FBXW7 gene 310. Conversely, some mutations may be deleterious, killing the cells by themselves or by working with other harmful single nucleotide polymorphisms (SNPs), since about 12% of the SNPs are harmful in the human genome 311, especially in Europeans 312. Actually, since the most common genetic changes found in tumors are large chromosomal deletions 313,314, severe genomic damage may lead to the loss of those genes required for cell survival. Moreover, some pernicious mutants may undergo mutation again, back to the wild type or to a better version, which may have reverse evolution as its essence 315 and cause differentiation of the cells. This so-called “back mutation” or “reverse mutation” is occasionally discerned in drosophila 316, as well as in some human genetic diseases 317-320 and in some cancers treated with chemotherapeutic agents 321-323.

Inauthenticity of the tumors may be another reason for spontaneous regression. For instance, it was often reported in the 1970s-1980s that hepatomas and hepatocellular carcinomas in women chronically using estrogen-rich oral contraceptives regressed upon termination of the contraceptive use 324-330, which substantiates the human relevance of estrogen-induced hepatomas in rodents reported in the 1950s-1960s 331-333. As another example, low-grade lymphomas can result from infection by Helicobacter pylori (HP). These tumors are basically curable by eradication of the bacteria with antibiotic treatment 334-339 but, if left untreated, some of them will progress and become incurable, as reviewed by Park and Koo 340. Similarly, Chronic HTLV-I (human T cell lymphotropic virus type I) infection may spawn adult T cell leukemia or lymphoma, but the neoplasm can be well controlled or even cured by antiviral treatment against HTLV-1 341-343. To us, these properties of these estrogen-, bacterium-, or virus-caused outgrowths resemble those induced in many animal models described above, and thus are not authentically neoplastic at their early time point although their diagnoses meet pathological criteria for neoplasms and they, if left untreated, may eventually evolve to genuine neoplasms. Or, we can take a non-pathological definition of cancer proposed by Robert Axelrod, who majored in political science but became a prominent cancer ecologist 344, that incipient cancer cells might just have been partly transformed, and not yet fully malignant, thus requiring collaboration with each other for survival and for collective presentation of a cancer phenotype 345.

Using very strict criteria, there may not be pure spontaneous regression or remission of cancer in humans, because it is unlikely that patients will do absolutely nothing for their illness. Some patients' self-management towards the neoplasm may actually be effective, although their doctors may not realize it. The patients may have experienced severe infection, especially a febrile one, since an infection or fever may be an effective cancer remedy partly by enhancing the immune attack on the cancer cells 256, 346-367, as we have reviewed before 296, 368. Moreover, regression may occur via an unknown mechanism, such as via spontaneous epigenetic or genetic changes leading to a full differentiation of the tumor cells 369-371 or increased stimulation of immune function by the tumor cells 372, 373.

### Tissue culture and transplantation were once used to determine immortality and autonomy

Even over a century ago, whether or not a patient's tumor was immortal and autonomous had been a concern of, and thus had often been tested by, surgical pathologists, because they had realized that morphological traits should not be the solitary criterion, and the tumor's behavior should also be considered, for an infallible diagnosis of cancer. The tests had been conducted, ever since 1901 374,375, mainly with culture of surgically removed tumor tissues or with transplantation of the tissues to animals, the two modern techniques aforetime. Actually, a technique involving both transplantation and culture was done by inoculating tumor cells into a fertile egg and then hatching it 376-386, which is the parentage of some modern chick embryo assays for cancer research 387-399 such as the chick heart invasion assay 400-405. The rationale for using tissue culture is that neoplastic cells are immortal and can self-renew to forever maintain themselves as a “new organism” by incessant cell division. Even after the patient has died, the “organism” can be maintained as cell lines, embodied by the Hela cell line established in 1951 from cervical cancer of the late patient Henrietta Lacks 406.

Human tissue transplantation to animals, started by Peyrilhe in 1773 with cancer fluid 5 and by Hanau in 1889 with solid tissue 407, has been overwhelmingly used in cancer research, as extensively reviewed even many decades ago 407-422. Mention should be made of the studies over a century ago that involved tumor transplantations to humans 423-425, with the heroic trial by Senn who inoculated himself with pieces of cancerous lymph nodes 7, 425. Moreover, as reviewed by Triolo 7, transplantation of animal tumors to other animals have also been performed since in 1860s 426-430. The rationale for this approach is to use tumor cells' behaviors, mainly autonomy, to determine its authenticity. As shown in table 1, transplantation of animal tumors can generally be divided into five categories 305, 431, i.e. 1) autologous transplantation, or transfer back elsewhere in the same animal; 2) homologous I transplantation, or transfer to a tumor-bearing animal of the same species; 3) homologous II transplantation, or transfer to a normal animal of the same species; 4) heterologous I transplantation, or transfer to a tumor-bearing animal of a different species; and 5) heterologous II transplantation, or transfer to a normal animal of a different species.

A seminal finding by Greene in the 1940s, among his many other findings 305, 431-438, is that some cancers are not transplantable to normal animals but are transplantable to the animals that bear a spontaneous tumor, especially one of the same tissue origin 305, 431. For instance, the Brown-Pearce rabbit tumor typically does not grow in normal C3H mice but it grows rapidly in those bearing spontaneous tumors 433, and a Rous chicken sarcoma grows well subcutaneously in tumor-bearing C3H mice but not in normal C3H mice 305. These results led Greene to a conclusion that the factors affecting the take of transplanted tumors “are constitutional in distribution and are not localized at the site of the primary growth” 432. However, lymphoblastic leukemia and lymphosarcoma are graftable to every normal genetically compatible host but do not produce tumors in the anterior chamber of an eye of an alien host, showing a difference from other tumors 305. The difference between normal and tumor-bearing hosts in response to a tumor graft suggests that tumor-bearing animals possess some factors affecting the graft's survival. A plausible interpretation is that the spontaneous tumor preexisting in the host has already suppressed the host's immune function that is supposed to reject the graft. Studies of these inhibitory effects have later been extended to the interaction between normal cells and tumor cells not only *in vivo* but also *in vitro* 439-448, as has been reviewed by us 449, by Rubin 450-453, by Aktipis 454,455 and by Thomas et al 456-458 from different slants. For instance, it has been shown that normal cells suppress the growth of adjacent tumor cells in culture 459 and in skin grafts on mice 460. Unfortunately, identifying these tumor or host factors has largely been neglected, although it is important since manipulation of these factors may be helpful in curing cancer.

Another trailblazing finding by Greene et al. in the 1940s is that the tumors that are capable of metastasizing are heterologously transplantable, as they can grow in the brain or the anterior chamber of an eye of animals of a different species, whereas tumors that are still incapable of metastasizing cannot 431, 435, 437, 438. Based on these observations, Greene concluded that only those lesions which can metastasize are fully autonomous and can be regarded as cancers, whereas those which do not possess this ability are still conditionally autonomous and thus should not be regarded as malignancy 435, 437. Although in pathology textbooks metastasis is not a canon for diagnosis of a malignancy, it is the only reliable yardstick to distinguish malignant neoplasms from benign ones 461. Considering that even today, compared with Green's epoch, in the surgical pathology service we still do not have a simpler or more reliable approach to determine whether a primary tumor removed from a patient has encompassed the ability to metastasize, it is a pity that Greene's simple but reliable test has not been used in clinical service until now, probably due partly to an ethical concern on the eye graft.

### Most animal models have not yet been tested for the trajectory of “induction, reversibility, and progression”

Many animal models of carcinogenesis induced by chemicals or hormones have not yet been determined for the inducer-dependency. Even worse, except the several models described in an above section, like the ones described by Sanchenz-Garcia's group 220,462, the vast majority of genetically manipulated animal models have not yet been tested either. This severe defect is presumably ascribable to two reasons: first, probably many molecular biologists have not realized that immortality and autonomy are prerequisite criteria for neoplasms. Second, the genetic manipulation in many, probably most, of these animal models is not set in a “turn-on/turn-off” mode, and thus does not allow researchers to control the target gene to determine whether or not the lesions are inducer-dependent. Moreover, for the induction of visceral tumors, like the N-nitrosobis(2-hydroxypropyl)amine-instigated lung tumors 463, the determination is more difficult as it requires sacrifice of the animals. We surmise that most of the undetermined animal models may also show an inducer dependency until a late stage, with their carcinogenic procedures following the aforementioned trajectory of “induction, reversibility, and progression” described by Rusch 71. Considering that the lesions wrought by c-myc and mutant k-ras, the two most potent oncogenes, already manifest such dependency, other genetically manipulated models will likely show this trajectory as well. Notwithstanding, this conjecture needs to be substantiated by studying untested animal models, especially the new ones to be established in the future using, for example, a conditional transgenic or knockout approach.

### Loss of allegiance to the host's body is the essence of neoplastic cells' immortality and autonomy

Sporadic tumors can be derived only from those cell types that are renewable, i.e. have a lifelong ability to replicate, because mutation needs to be perpetuated by at least one round of DNA replication and to be passed to filial cells via cellular divisions 155, 449. That permanence becomes possible because the fitness testing of cells is usually conducted after the mutation is made permanently heritable 464. We tag those highly renewable cell types as “anabolic” for their great susceptibility to cancers and those that have lost their replicative ability in adulthood, such as neurons and cardiac myocytes, as “catabolic” for their role in the development of type 2 diabetes 465. Even for those renewable cells, it will take about one-fourth to one-third of the lifespan to complete the procedure of sporadic carcinogenesis, which is about 20-30 years for human beings 306, 371, although it could take 50 years by others' estimation 19. Therefore, the aforementioned tumors induced by 3-methylcholanthrene in just 3-4 weeks cannot be authentically neoplastic 64, 65, since the lifespan of experimental mice and rats is three years or longer 205, although their counterparts in the wild live much shorter lives 24. Indeed, we are not aware of any rodent model in which a sporadic cancer can be induced in a period less than a few months, except those genetic models in which the genetic manipulation has already been effective during an embryonic stage, thus mimicking a pediatric (but not a sporadic) carcinogenesis, as to be expanded upon later.

All cell types in an evolutionarily complex animal have a physiological total number. For renewable cell types, if the cell number is decreased for some reason, the body will trigger cell proliferation to restore the physiological number. Conversely, if the number is higher than normal, as seen in over-regeneration that often happens following a regeneration procedure, the body will goad some of the cells into apoptosis to avoid cell redundancy 296, 302, 303, 466-469. This is because apoptosis evolves as a specific mechanism to eliminate useless, redundant cells from the tissue or organ 302, 303, 466, 468, but not as a demise mechanism triggered by compensatory proliferation as thought by some peers 470-473. Indeed, compensatory proliferation is regeneration and does not aim to engender excessive, i.e. hyperplastic, cells, although it usually does mildly because of a slight overproduction of cells. Killing excessive cells via apoptosis can be implemented in an evolutionarily complex animal because all cells have allegiance to the animal's body, as we described before 296, 302, 303, 466-468, or “conform with the law of organisms”, as put by Rous in 1941 58. This allegiance as the “law of organisms” allows the host's body to require some renewable cells to sacrifice their lives for the body's ultimate interest. An instructive example is that white blood cells are often put on the frontier by the host's body to fight against infectious micropathogens and die in the battle, so that the host as a whole can survive 466, 474. However, sometimes some renewable cells, such as select bone marrow cells, epidermal keratinocytes, and mucal cells in the gastric-intestinal tract, have lost their altruism, usually due to acquisition of tumor-driving mutations that make the cells egocentric. These selfish cells want to survive stress such as micropathogen infection, over-regeneration-trigged apoptosis, etc., and become independent of the body, i.e. become autonomous. Reiterated, this loss of loyalty to the host's body is the essence of, or the reason for, autonomy of some cells. “Fail to conform with the law of organisms” as said by Rous 58, or “become autonomous” as outlined by Ewing to be the pathological concept of a tumor 475, was set as “the signature of a genuine neoplasm” by Borst in 1903 476 and has, until today, been a salient feature of benign and malignant tumors.

In addition to apoptosis, an accelerated aging procedure leading to senescent death may be an additional mechanism for elimination of the excessive, i.e. hyperplastic, cells in the early lesions of animal models; although studies on the mechanism for the inducer-dependency have hardly been extended to this type of cell death. We define cell death via aging as “senescent death” 302, because normal cells have their lifespans 477-482 and ever since it was first observed in 1965, this senescent phenomenon has immediately been linked to aging 483, 484. Indeed, a host of studies have shown that aging and senescence are highly interrelated 24, 484-497, although senescence itself is defined as a permanent growth arrest that does not necessarily lead to death of the cell 487, 491-493,498. Senescent death is also an evolutionarily developed demise program, but unlike apoptosis, it aims to eliminate those aged, although still useful, cells 302, 303, 466.

### Hyperplasia is the responsive type whereas neoplasia is the intrinsic type of growth

Hyperplastic and neoplastic cells differ starkly in not only their cell death pattern but also their growth pattern. Leslie Foulds (1902-1974) split growth rate into “the responsive” and “the intrinsic” components, with the total growth of the cells being the sum of the two 155. He wrote in 1953 that, “all cells which can give rise to cancer possess the ability to multiply at a given rate, provided the environmental conditions are constant. They also have the capacity to respond to nutritional and hormonal growth factors, temperature, pH, etc. The intrinsic growth rate of normal cells is in general low; their responsive growth rate is high. The cancerous change goes with acquisition of a greater intrinsic growth rate and diminished responsiveness; the more malignant a cell, the greater the intrinsic and the less the responsive growth” 155. In today's language of cancer research, “the responsive growth” is the regenerative type of cell proliferation that is controlled by the host's body 466 and dwindles away during carcinogenesis, whereas “the intrinsic growth” is the autonomous proliferation that is controlled by the cells themselves and is strengthened during carcinogenesis. Hyperplastic cells are still loyal to the host's body and thus their growth belongs to the “responsive” type.

### Autonomy is manifested not only as uncontrolled replication but also as uncontrolled function

Although a neoplastic nature is defined as “uncontrolled replication” attributed to the gain of intrinsic replicative ability, in reality there are some tumors that do not actually kill patients by expansive cell proliferation but, instead, by their uncontrolled functions 499. Examples include some endocrine tumors, such as some islet-cell carcinomas that secrete insulin 500 and pheochromocytomas that secrete catecholamines such as adrenalin 501. As the most salient feature of these tumors, the patient's body has lost its control over the tumor's functions. While the tumor is still small without invasion or metastasis, a virulently high level of the hormone it secreted may have already killed the patient. Keloid scar, which is not classified as tumor in pathology textbooks but show neoplastic features such as recurrence and incurability, may be an example of uncontrolled function of benign lesion 502-504, as its fibroblasts constantly produce collagen. Moreover, uncontrolled function may sometimes show as uncontrolled metabolisms, embodied by such as cachexia-incurring cancers that elicit high metabolic rates to cannibalize many cells of the patient for energy. Therefore, disloyalty to the host's body can be manifested mainly as the loss of the host's control over the tumor's functions or metabolisms, and not predominantly as the loss of the control over the tumor's cell proliferation, as Markert 499 and Pitot 505 had already pointed out in 1968.

### Animal models can generally be dichotomized

**Figure 1:**
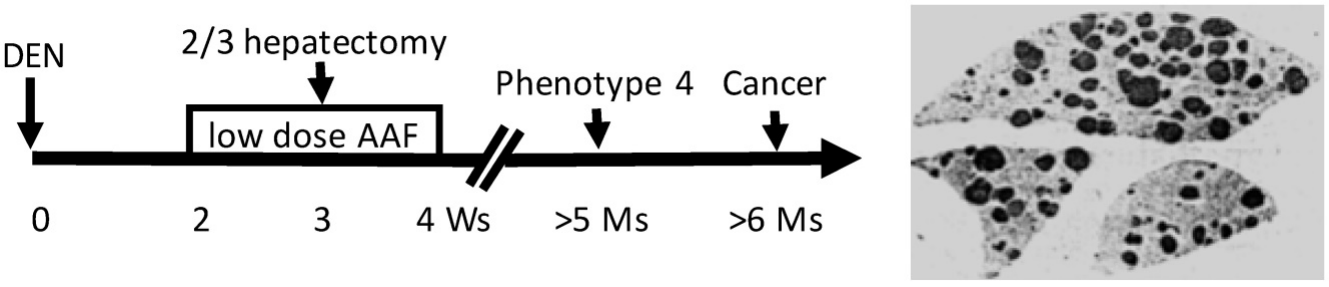
The Solt-Farber's “resistant hepatocyte” model of liver carcinogenesis in the rat. A toxic dose of diethylnitrosamine (DEN) will 1) cause liver necrosis and 2) create initiated hepatocytes. Two weeks later, when the liver has recovered from the necrosis, the rat will be given a low dose of 2-acetylaminofluorene (AAF) for two weeks, function of which is to inhibit proliferation, so-called mitoinhibition, of hepatocytes, but the initiated cells are resistant to this inhibition. In the middle of AAF treatment, hepatectomy will be performed to remove two-thirds of the liver, which provides a strong impetus for regeneration. Because normal hepatocytes are mitoinhibited, all regeneration pressure is imposed onto the initiated cells, driving them to proliferate robustly and form nodules. The image at the left shows these nodules visualized by immunohistochemical staining of the P form of glutathione S transferase, a marker for the nodular cells, in the three remaining lobes of the liver four weeks post cessation of AAF treatment 201,852. These nodules will regress afterwards but some new focal cells, which can proliferate spontaneously and are coined by Farber as “phenotype 4”, will later develop from some of the nodules 507,508. One or several of these phenotype-4 lesions will eventually progress to overt cancers.

Animal models established since the 1900s have evolved using, as the inducer, a single agent to using a complex regimen or manipulations. Nevertheless, we try to split all animal models into two groups, based on whether or not the inducer is a potent genotoxic agent, although there are many intermediate models in which the inducer is a combination of both genotoxic and non-genotoxic agents 506. In one group wherein the inducers are potent in causing mutations, mutation(s) responsible for the initiation occur early. A prime example is the Solt-Farber's “resistant hepatocyte” model of hepatocarcinogenesis in the rat (Fig. 1) 507, 508, or our modified version of it in which the promoting agent 2-acetylaminofluorene is routed via gavage instead of by feeding *ad libitum* 509-512. Carcinogenesis in this group follows a trajectory of “initiation-promotion” or “initiation-promotion-progression”, as detailed by Farber 39-41, 513, 514. It is clear that the genes and their mutations responsible for initiation are not those responsible for immortality and autonomy. This can be discerned in the Solt-Farber model wherein spontaneous proliferation, which reflects immortality and autonomy, occurs only in the lesions coined by Farber as “phenotype 4” that appear months after the establishment of initiated cells and after the completion of the carcinogenic regimen (Fig. 1) 514.

The other group of animal models uses non-mutagenic agents as the inducers, which in the literature are often dubbed as “epigenetic carcinogens or agents” 515-520, “nongenotoxic carcinogens” 519, 520, or “cocarcinogens” 34, 521-523. In our opinion, carcinogenesis in this group often incepts with promotion, but not with initiation, unlike that in the aforesaid group. This is because the nongenotoxic inducer in this group kindles proliferation of normal cells without incurring mutation(s) or even epigenetic aberration(s) to establish initiated cells in the early incarnation, and therefore the early proliferative lesions are not of initiated cells, meaning that initiation with some genetic changes, and the subsequent neoplastic transformation, occur much later in this group of models than in the above one. Alternatively, initiation in this group of animal models might not involve mutations, as considered by some investigators 307, 524-531.

### Unlike in animal models, immortality and autonomy may occur early in most human tumors

**Figure 2:**
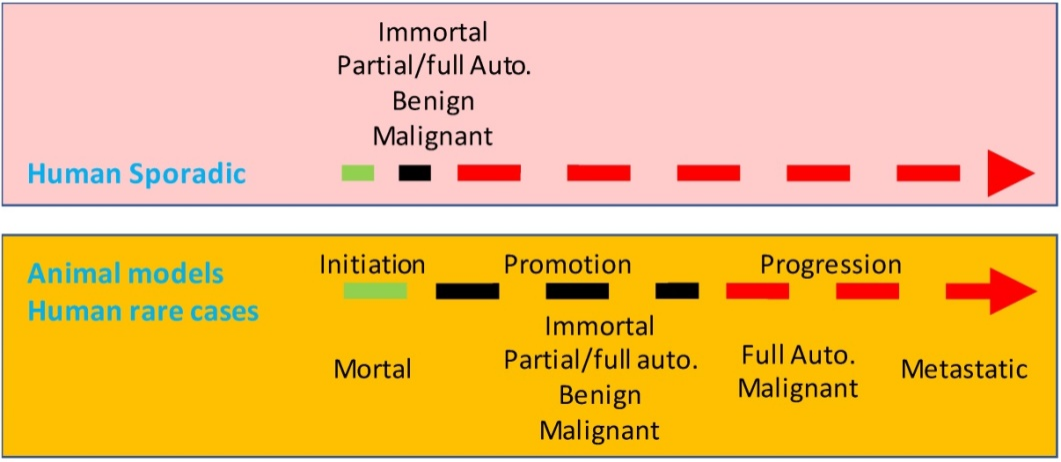
Illustration of a speculative difference at the time point for the establishment of immortality and autonomy between the tumorigenesis in most animal models and that in most human situations. In humans, immortality (Immort.) and autonomy (Auto.) may occur at a very early time point, thus establishing small lesions as genuinely benign or malignant neoplasms. In contrast, tumorigenesis in most animal models is a stepwise procedure of initiation, promotion and, in some cases, progression as well. Initiated cells are still mortal and thus are not neoplastic. Immortality and autonomy in animal models occur at late promotion or at the progression.

When are immortality and autonomy established during a lengthy tumorigenesis in humans? It is an enthralling brainteaser, so far without an answer 532. For several reasons we infer that in most cases they occur at an early time point (Fig. 2). First, spontaneous regression of tumors is rare, and thus nearly all tumors, many of which are diagnosed at early stages, are considered immortal. Second, in our pathology service and in the literature 533, we occasionally encounter very tiny malignant tumors in patients. Albeit the small tumor had already been surgically extirpated or considered cured, some patients still died of its metastasis years later 534, which substantiates the malignant authenticity of the small primary tumor. Third, autopsies of humans that died of various causes found about 3-27% of the bodies had an occult pituitary adenoma 535-539 (and DJ Liao's empirical knowledge), and magnetic resonance imaging of normal human volunteers found this tumor in about 10% of normal persons 540. Similarly, it has been known since 1934 that a large number of men over 40 years of age have occult prostate adenomas or adenocarcinomas, although many of the lesions do not develop to clinical cancer before the men die from other reasons 541-546. A much higher incidence of occult tumor occurs in the thyroid, since one early report showed that 49.5% of 821 clinically normal people contained nodules, 17 of which were histologically malignant 547,548. Similarly, unselected autopsies of children before three months of age also found neuroblastomas in the adrenals at a frequency 40-50 times higher than the reported incidence of this tumor 549. Fourth, as summarized by Blagosklonny 532 and Kolquist 550, even many premalignant lesions in humans show immortal traits, such as elevated expression or activity of telomerase. Nevertheless, more tangible proof for the speculative early-establishment of immortality and autonomy is still needed.

In humans, tumor-promoting momentum is much weaker, including the impetus provided by those relatively potent promoters such as cigarette smoking or chronic viral hepatitis, compared with that provided in various animal models. Therefore, human lesions grow and progress much more slowly, allowing immortality and autonomy to occur much earlier with respect to the size of the lesions, and allowing the neoplastic transformation to occur as the result of some relevant mutation(s), long before the patients feel something wrong and go to see their doctors. This is partly because a lengthier course allows accumulation of more haphazardly-occurring mutations, including the one(s) required for immortality and autonomy, if we accept the notion that tumors, especially cancers, occur as repercussions of mutations that have cell-autonomous modes of action 29-31, 525, 526, 551-558.

The inducer-dependency of the early tumors in most, if not all, of animal models indicates a very late establishment of immortality and autonomy, which collides with the perceivable early-establishment of immortality and autonomy in human lesions described above. In other words, few, if at all, animal models established so far reflect the tumorigenic course in most human situations (Fig. 2). Fortunately, in some rare human situations, immortality and autonomy are likely to be established in a late stage. For example, familial colorectal polyps that will sooner or later progress to cancer are developed due to inherited mutations in some genes, like the APC (adenomatous polyposis coli) gene 559-563. The constant presence of the mutation serves as a lasting coercion on colorectal mucal cells, keeping them in an unremitting state of proliferation to form polyps. These polyps are considered in pathology as premalignant lesions, pursuant to their morphology and to the fact that cancer likely ensues. Notwithstanding, we are curious about whether the polyps would regress if we have a way to correct the mutation, since spontaneous regression not only of polyps but also of small colorectal adenomas has been well recognized by pathologists 564-566. Probably, from the point of immortality and autonomy, the still-mortal polyps are “preneoplastic”, an elegant jargon used by Haddow 72 and Rubin 567, or are “precursor lesions”, another good appellation used by Farber 568. Other embodiments of the late establishment of immortality and autonomy in human outgrowths include the abovementioned curable hepatomas and hepatocellular carcinomas caused by chronic use of oral contraceptives 324-330, and lymphomas or leukemia caused by HP 334-340 or HTLV-1 341-343. Some thyroid tumors may also be mortal and may not evolve to authentic neoplasms in the patients' lifetime 569, which dovetails with Forth's opinion in 1953 155.

### What do the two genetic hits do in carcinogenesis, and is a third hit needed?

**Figure 3:**
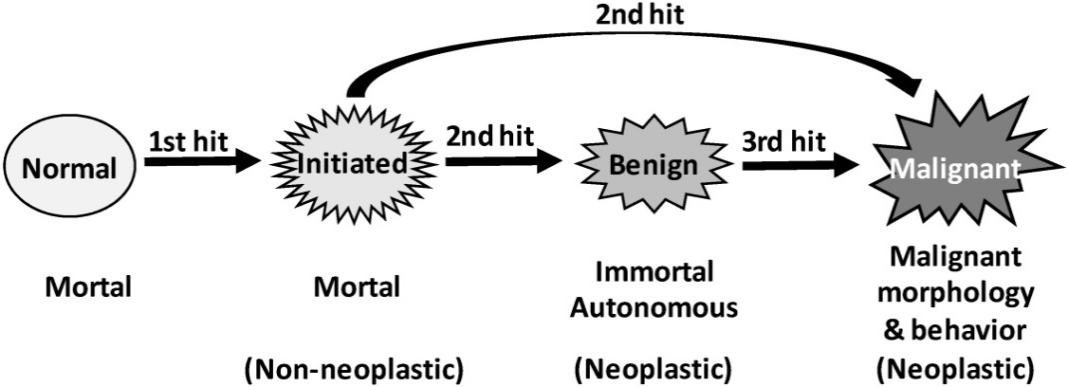
Illustration of our three-hit hypothesis. Coupling the traditional two-hit principle with the initiation-promotion theory leads us to a supposition that the first genetic hit establishes initiated cells that are still mortal and non-autonomous, whereas the second hit creates immortality and autonomy, thus establishing neoplastic cells, either benign or malignant. Since formation of benign neoplasms also requires two genetic hits, we extrapolate that, in some animal models and probably also in many human situations, establishment of malignant morphologies and behaviors requires a third hit on the relevant gene(s).

Tumorigenesis may sometimes occur via only “one-hit” 570-572, but “two hits” are usually required 17-20. Although the “two hits” are still ill-defined, sometimes as two genetic alterations but some other times as “initiation” and “promotion”, the concept accepts the century-old ideas that carcinogenesis results from genetic alterations and that cancer cells owe their properties to mutations 450, 573-576. We mingle the two different “two hits” definitions together and consider that the first genetic hit is for creation of initiated cells that differ from their surrounding cells in response to promoting environment (Fig. 3). According to Farber 39-42, 513, 514, 568, 577-582, in most cases promoting agents cause “mitoinhibition”, i.e. inhibition of mitosis or proliferation, of normal cells, whereas initiated cells are resistant to this inhibition (Fig. 1) 200. Actually, a condition disfavoring cell growth in cell culture, such as a lower serum concentration or a cell confluence situation, is an impetus to drive neoplastic transformation as well 313. Therefore, in a promoting environment, probably also in humans 313, only initiated cells can robustly proliferate to form lesions, especially when many of their adjacent normal cells die and the organ or tissue has a strong demand for regeneration 200, 201, 583. This “mitoinhibition” theory conforms with the hypothesis of Rozhok and DeGregori that cancer occurs more often in old age 453, 584, because normal cells in the elderly, compared with their counterparts in the young, have less proliferative capacity, thus being more “mitoinhibited” and providing the spontaneously-occurring initiated cells with a stronger promoting momentum 24. The molecular mechanisms of promotion via mitoinhibition still remain enshrouded. We extrapolate, with trepidation as sans evidence, that mitoinhibited normal cells promote proliferation of initiated cells in part via a mechanism similar to that used by senescent cells to promote carcinogenesis of their adjacent cells, since senescence is a state of permanent growth arrest 487, 491-493, 498, i.e. “permanent mitoinhibition”. This mechanism is coined as SASP (senescence-associated secretory phenotype) 585-587, and its effect on carcinogenesis has been extensively reviewed in the literature 487, 588-596.

In Rubin's punditry, the cells of skin papilloma produced in the aforementioned animal models that regress upon withdrawal of the inducer are initiated 451, which connotes that initiated cells are not immortal. Indeed, in Farber's “mitoinhibition model” of hepatocarcinogenesis described above, most initiated cells in the focal lesions eventually die of apoptosis 39-42, 513, 514, 568, 577-582. In our meditation, the second hit converts initiated-cells into a neoplastic state, benign or malignant, by rendering the cells immortal and autonomous (Fig. 3). This second hit occurs in a later promotion stage of the “initiation-promotion” models or in the progression stage of the “initiation-promotion-progression” models. In sporadic carcinogenesis in humans, initiated cells may also exist, although they are technically difficult to identify. Nevertheless, “preneoplastic” cells in humans may have already experienced the first hit, while “pre-cancerous cells” may have also experienced the second hit.

In some carcinogenic procedures wherein a malignancy does not require a benign lesion as a precursor and thus a second hit is sufficient, the mutation(s) responsible for immortality and autonomy may also be responsible for malignant morphologies and behaviors (Fig. 3). However, in other animal models and in human situations, the mutations responsible for establishing immortality and autonomy may not be the ones responsible for establishing malignant morphologies and behaviors, since benign neoplasms have also experienced the second hit. Therefore, in these situations a third genetic hit may be required to establish malignant morphologies and behaviors (Fig. 3). Of course, malignant neoplasms continue to evolve via many subsequent hits to be more and more heterogeneous and heinous.

### An old, but still unanswered, question is how many mutations are needed for completing a carcinogenesis

The target or targets of the abovementioned two or three genetic hits remain unknown to us. Initiation created by the first hit likely involves only one or several genes, since initiated cells are morphologically indistinguishable from uninitiated ones 40, 42, 514, 597. Immortality and autonomy created by the second hit may involve only one or several genes as well, since many benign tumor cells, such as uterine leiomyoma cells, are quite similar to their normal counterparts in cellular morphology. Therefore, it is not surprising that acquisition of immortality does not require genetic instability, and cancer cells can be created and sustained without gross genetic changes 598-600, although instability and gross mutations can occur even at an early time point of carcinogenesis 601. The inference that only one or several genes are involved is also supported by the fact that immortalization of a mortal cell to establish a cell line has been proven to be easy, especially *in vitro* 139, 602-608. For instance, targeting both the p16ink4 and c-myc genes can immortalize human mammary epithelial cells invitro 598, and the IgEGF and SV40T bi-transgenes can immortalize murine cells 609. Actually, immortalization is easier when the cell has a small-rodent parentage. Simply knocking out the p53 gene alone can immortalize mouse hepatocytes 609, and even ectopic expression of a 3'-untranslational region of a gene without expression of the protein 610 can immortalize rat embryonic cells. A so-called “3T3 protocol”, mainly transferring 3x10^3^ cells from a flask to another every three days, had been established almost six decades ago as an effective procedure to immortalize mouse cells, especially embryonic ones 611-613. This simplicity is presumably because small-rodent cells have their telomerase constantly “on” and have only a single barrier to immortalization controlled by the RB (retinoblastoma protein) pathway 602, 614, 615.

The third hit, if it is needed, may also require only a small number of genes, in our opinion, since the second hit can do both, i.e. can immortalize the cells and confer malignant morphology and behavior upon the cells. Therefore, the sum of the two or three hits may be congruent with the estimation by Hahn and Weinberg that five alterations are required for converting human cells to malignant phenotype 616, or by Armitage and Doll in 1954 31 and by Vogestein in 1993 617, that carcinogenesis requires only six or seven mutations. Fluid cancers such as leukemia may require even fewer and thus may be relatively easier to cure, generally speaking, as we inferred before 449. A caveat is that different hits in different cases may involve different genes, especially for the third hit that is responsible for cellular and histological morphologies and behaviors that can vary greatly among different cases of the same cancer type. This variation makes the sum of “initiator genes”, “immortalizer genes”, or “malignant morphology responsible genes” large, and the sum of all three even larger, which is a major reason why there have been a huge number of genes found to be cancer-relevant.

### Actually, whether mutation is needed or not for tumor formation is still debatable

**Figure 4:**
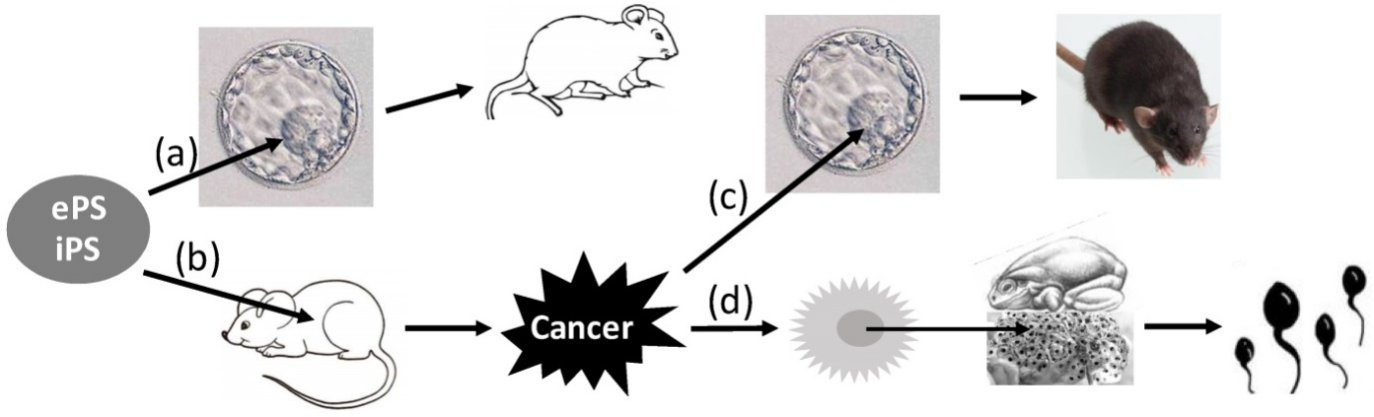
Reversion of pluripotent cells between normal and cancers. Embryonic (e) or induced (i) pluripotent stem (PS) cells introduced into the blastocyst in the uterus can develop to live animals (a). However, if the cells are transplanted to extrauterine sites of adult animals, they will likely develop to teratomas or teratocarcinomas (b). If teratocarcinoma cells are inoculated into the blastocyst, they will be incorporated into the developing embryo, and the tissues of the animal developed from the embryo will be chimeric, i.e. containing cells from both the embryo and the cancer (c). Moreover, if inoculation of the nuclei isolated from the Kucké renal cancer cells of the frog origin into enucleated frog eggs, the eggs can hatch out live tadpoles with all tissues normal (d).

Although it has become “Tumor 101” that tumors are caused by variegated genetic alterations, collectively coined herein as “mutations”, there has always been a theory considering that mutations are not necessarily required, which, since it was broached by Rous in 1947 531, has continued receiving supportive laboratory data 307, 525-530, 618-621. Some peers even consider that heterogeneity of cancer cells may not necessarily be related to the increases in mutations either 622. A major piece of evidence supporting this non-mutation theory is from Stevens' reports in the 1960s, which showed that transplantation of germinal stem cells from early male mouse embryos of the 129 strain to testicles of adult mice led to the development of teratoma or teratocarcinoma (Fig. 4) 623-625. As reviewed by Buta, Bustamante-Marin, Damjanov, Arechaga, Blum, Sell, Martin, and Pierce 4, 626-639, many other researchers also reported later that early embryonic cells, including those of human origin 630, placed into several extrauterine sites of adult animals could develop to teratoma or teratocarcinoma 629, 640-644. A slew of studies in the past decade have extended these findings by showing that induced pluripotent stem cells transplanted to animals can develop to teratoma or teratocarcinoma as well (Fig. 4) 626, 645-650. Considering that extrauterine sites should not be mutagenic, these observations support the non-mutation theory. Moreover, this tumorigenesis involving embryonic or induced pluripotent stem cells can be minimized or prevented by different manipulations 626, 647, 648, 650-652. Conversely, teratocarcinoma cells injected into the blastocyst can be incorporated into the developing embryos, and the organs or tissues of the mice developing from such embryos contain cells from both the blastocyst and the cancer (Fig. 4) 653-661. More convincingly, injection of the nuclei isolated from the Lucké renal cancer cells of the frog origin into enucleated frog eggs allows the eggs to hatch out tadpoles that are normal without any trace of cancer (Fig. 4) 285, 662-668. Similar conversion back to a normal state by an embryonic microenvironment has also been shown for a few other cancer cell types 448, 669-673, 673, 674. For instance, with models of chick embryo and Zebrafish embryo as well as with intrauterine injection approach in mice, many studies have shown that human malignant melanoma cells in an embryonic microenvironment do not develop to tumors but, instead, differentiate to neural-crest-like cells 675-678. Some cells of squamous cell carcinomas have also been observed to differentiate into mature keratinized cells as squamous pearls 679. Therefore, as pointed out by Pierce in 1974 632, the concept of “once a cancer cell, always a cancer cell” may not always be correct. Because mutations are unlikely to disappear by themselves 632, development of mature tissues from cancer cells lends color to the non-mutation theory.

It is worthy of mentioning that the abovementioned experiments with teratocarcinoma cells have a much better version in plant tumor systems, as thoroughly reviewed by Braun four decades ago 680. It has been shown in several plant species that teratomas can be reverted to normal plant cells and that tumor cells grafted to another plant can develop to a normal plant that can bloom and produce seeds, and the seeds can germinate and grow to normal plants 20, 681-687.

**Figure 5:**
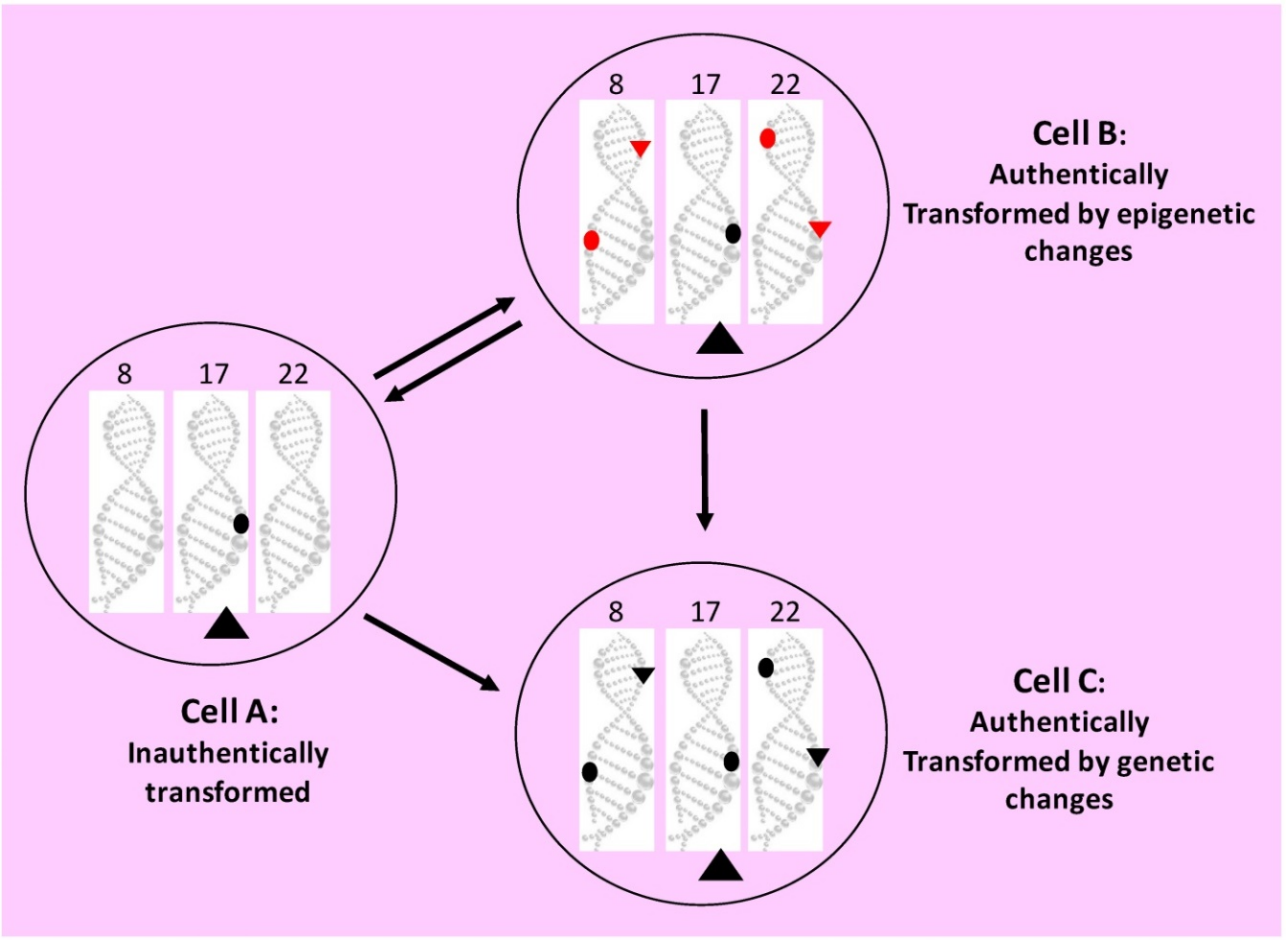
Depiction of the “coercion hypothesis”. Our manipulation, say as a transfected cDNA (the large black triangle in the cytoplasm of Cell A) or as a transgene or a gene-knockout on the chromosome 17 (black dot in Cell A), coerces the primary cell into incessantly replicating and manifesting transformed morphology or behavior, such as colony formation in agar. The relentless proliferation will eventually lead to spontaneous occurrence of the epigenetic (red dots on the DNA of chromosomes 8 and 22 in Cell B) or genetic (black dots on the DNA of chromosomes 8 and 22 in Cell C) alterations that establish immortality and autonomy, making the cell truly neoplastic in behavior. Continuous proliferation will also cause spontaneous occurrence of the epigenetic (small red triangles on the DNA of chromosomes 8 and 22 in Cell B) or genetic (small black triangles on the DNA of chromosomes 8 and 22 in Cell C) alterations that establish neoplastic morphology. This is to say that immortality and autonomy as “the behavior aspect” of neoplastic property, as well as “the morphology aspect” of neoplastic property, may sometimes be controlled separately by different sets of epigenetic or genetic alterations, i.e. different sets of “hits”. Moreover, the cell authentically transformed via epigenetic mechanisms (Cell B) may initially be reversible back to the normal, but later it will likely develop such genetic alterations that make the cell lose the reversibility and progress into the state of Cell C. If our manipulation is made in a controllable manner and is withdrawn early, the primary cell (Cell A) will no longer manifest the transformed morphology and behavior and will undergo senescent death (if the cell is in a culture dish) or both senescent death and apoptosis (if the cell is in a live animal). However, the truly transformed cells (Cells B and C) may retain their neoplastic properties sustained by the epigenetic or genetic alterations, unless some extrinsic factors (such as a chemical) cause the cells to circumvent or override the epigenetic or genetic alterations and make the cells reverse back to the normal state with or without retaining the alterations. In other words, neoplastic morphology and behavior incurred by our manipulation, an extrinsic factor, are inauthentic, but those caused intrinsic epigenetic or genetic alteration(s) are authentic.

Another major piece of evidence invigorating the non-mutation theory comes from the studies showing the reversibility of transformed cells back to the normal in cell culture 446, 453, 620, 688-691 and in animals or humans 197, 370, 692-695, with or without induction by chemicals 696-704. Actually, this reversion has received attention for almost a century 446,688,689, in part because direction of cancer cells to differentiation is a tantalizing strategy for cancer therapy. This reversibility is more clearly discerned in tumors of some species of fish and amphibians that are seasonal or temperature-sensitive, as aforementioned 277-285. However, the reversion does not necessarily indicate that the transformed cells were not initially transformed by mutations. It could be that the genetic alterations the transformed cells bear cannot prevent the reversion elicited by other genetic mutations or by some epigenetic changes. Or, alternatively, the extrinsic factors that cause the reversion can circumvent or override the initial mutations responsible for the neoplastic morphology and behavior (Fig. 5).

Epigenetic alterations certainly make a considerable contribution to the formation and progression of tumors 705. What we still wonder is whether these changes alone, without involvement of genetic mutations, are sufficient for the development of authentic neoplasms that are immortal, autonomous and irreversible (i.e. without undergoing spontaneous regression) and progress continuously towards more and more diabolical states, as seen in most cancer patients.

### Are immortality, autonomy, and transformation extricable from one another, and which occurs first?

In 1983 Land et al showed that embryonic fibroblasts expressing a ras mutant could form colonies in soft agar 706, which was shown by Freedman in 1974 as an insignia of a transformed state 139, 707. However, the transformed cells could not grow constantly and were still mortal, and their immortalization required concomitant expression of the c-myc gene or a viral oncogene 706. Similarly, expression of the SV40 large T antigen in mouse embryonic fibroblasts goads the cells into forming colonies in soft agar, but most of the cells eventually die 708,709. Primary cells concomitantly expressing a CDK4 gene and a ras mutant can form colonies in agar and develop to invasive tumors in animals, but the cells still cannot grow indefinitely in culture 710. All these data and others 711 strongly suggest that neoplastic transformation can occur before, and thus can be extricated from, immortalization, which is braced by the observations that telomerase itself is capable of prodding primary cells into growing in agar and in animals, independent from immortalization 532, 712 and transformation 371, 713. However, this seems to collide with the two-stage model that is sometimes perceived as that the “initiation” immortalizes normal cells whereas the “promotion” transforms the immortalized cells 370, 371. Newbold, Reddel, and some other cancer wizards also consider that immortality is an early and prerequisite step of transformation 477-481, 714.

While the above discrepancy still awaits an answer, a related question is raised as to whether autonomy can also be extricated from immortality, although this segregation collides with the facts that human cancers rarely regress spontaneously and that no human tumor shows this segregation. A keloid scar may be the only tumor-like lesion in the human we know that seems to show this extrication, as it shows functional autonomy by constant collagen production without showing clear immortality of its fibroblasts 502-504. Some animal models seem to show this extrication as well: epithelial cells have been shown to be evasive, disseminating, and able to enter into the bloodstream before they form primary tumors 715; and mammary epithelial cells can be manipulated to metastasize and colonize in the lungs before they are malignantly transformed 716, 717. Nevertheless, such separation is not discerned in some tests with traditional approaches involving chemical carcinogens, such as the “Syrian hamster embryo cell transformation assay” 718, 719.

In our opinion, if an *in vitro* study shows the above extrications, more-tenable proofs for the neoplastic state are needed. This is to say that once the cells are shown to be capable of forming colonies in soft agar, they need to be tested for immortality before we can announce that they have been transformed. Unfortunately, many published studies of *in vitro* transformation do not show this additional evidence corroborating the immortal nature of the cells.

### It is worth ruminating about why we can only induce several tumors in an animal

There is an obvious discrepancy between neoplastic transformation in cell culture and tumor formation in animals, which has been baffling us for a long time 200, 720: *in vitro* transformation assay usually results in a large number of colonies in soft agar. Because each colony develops from a single transformed cell, the appearance of many colonies means that many cells have been transformed; this in turn means that the transformation assay is very effective, although it is a short-time procedure. However, in most genetically modified animal models, each animal develops only several tumors in its lifetime, albeit the target organ or tissue of the animal, say the liver or the five pairs of mammary glands, have trillions of cells bearing the same genetic modification. For instance, only 1 of 10 mammary glands in a c-myc transgenic mouse develops a tumor 721, 722 and only 4 or 5 of 100 pancreatic islets develop β-cell tumors in a SV40-LT-transgenc mouse 723. Actually, most chemical-carcinogenesis models produce only one to several tumors per animal, to our knowledge. If evaluated with the number of tumors per animal as the criterion, the only plausible conclusion is that the efficacy of our *in vivo* transformation approach is negligible, as trillions of the targeted cells fail to be neoplastic. Researchers are usually content with the high percentage, sometimes 100%, of the manipulated animals that develop tumors, and do not ask why the remaining trillions of cells in the target organ or tissue of the animal, which received the same manipulation simultaneously, do not evolve to overt tumors.

### Many animal models are overpraised, due to neglect of the immortality issue

As aforesaid, evidence for immortality and autonomy has not been provided for the lesions resulting from the vast majority of genetically-modified animal models of carcinogenesis. More correctly, the time point has not been determined at which lesions in these animal models enter into the immortal and autonomous state. This is an uncomfortable but undeniable flaw of the relevant studies, although we should have been content with the profuse information provided by these models on the functions and underlying mechanisms of the genes manipulated. It is possible that many peers have already harvested the “cancers” from the animals for the mechanistic analyses before the lesions, probably large in size, have evolved to genuine neoplasms; likely due to the unawareness of the importance of the immortality and autonomy issue. In 1948, Greene emphasized that, “the problem of cancer is primarily a problem of behavior. A pathologist who examines tumor tissue under the microscope may observe significant details of form and structure, but he can never determine its malignancy from its appearance alone; only by its behavior in the living body can malignant tissue be unmistakably identified. Of two tumors with cells that look exactly alike, one may remain static or even disappear while the other inexorably spreads and kills the patient. Unfortunately many kinds and conditions of tissue which are not malignant bear a remarkable resemblance to cancer” 431. Because animal lesions, even if they are large in size, have not yet established immortality and autonomy, they can be cured easily, simply by withdrawal of the inducer or by chirurgic extirpation. This contrasts with the fact that most human cancers are not curable, at least not so easily. Moreover, “malignant” tumors in most animal models do not metastasize within the lifespan of the animals, whereas most human cancers will metastasize if untreated. Indeed, an early estimation has shown that fewer than 30% of genetically modified animal models of carcinogenesis produce metastases 724, although there are several models of mammary carcinogenesis showing metastases 725, 726. Some tumors from animal models can metastasize, but the metastases may still be inducer-dependent, embodied by the aforementioned metastases of the TSH-instigated thyroid tumors 155, 181, 185 and of the estrogen-induced renal tumors in hamsters 156-158. In a nutshell, the mortality and non-autonomy, the inducer-dependency, and the inability to metastasize are telltale evidence that many animal cancers are easy to cure and thus are disarming, which starkly contrasts with most human cancers. Moreover, among the species differences is that most spontaneous malignant lesions are mesodermal-originated sarcomas in mice but are epithelial-originated cancers in humans 727-729.

### We still have no way of directly transforming cells *in vitro* and *in vivo*

In the above sections we have described five phenomena that dissent from, and may threaten the bedrock of, the orthodox doctrine of how cancer develops: 1) transformation, immortality, and autonomy can be segregated from one another in the lab, and which one occurs first depends on the experimental design, although immortalization occurs first in the two-stage model. 2) *In vitro* transformed cells may still be mortal and reversible back to the normal; 3) formation of tumors may not necessarily involve mutations; 4) outgrowths induced in animals are inducer-dependent until advanced stages; and 5) neoplastic transformation in animal models may be perceived to have a negligibly low efficacy because each animal develops very few tumors after a long latency. All of these phenomena deliver us a single message: although numerous alterations have been identified in a huge number of genes, none of our *in vitro* or *in vivo* manipulations are able to directly cause the epigenetic or genetic alteration(s) required for the establishment of immortality and autonomy (Fig. 5). In animal models, there so far has not been any evidence showing that turning on or off a gene, which has been technically feasible for decades, can quickly turn on or off immortalized or autonomous features of the targeted primary cells. In cell culture, none of our manipulations of physical, chemical, or biological factors can quickly immortalize and transform primary human or animal cells. The targeted cells show immortal and transformed features only weeks later in the culture or months later in the animal, obviously as the events secondary to our manipulations. Exceptions do exist peculiarly, as some plant cells can be transformed after only 34-48 hours of manipulation 287, 730, 731; with a few more days of manipulation creating more aggressive cells 680, 732-739. Nevertheless, to our knowledge mouse or rat models require 6-9 months for the tumor induction, which is about 1/6-1/4 of the mouse or rat lifespan; although in some rare cases very potent chemical carcinogens might induce tumors in 3-4 months, i.e. about 1/12-1/9 of the lifespan. Considering that the early tumors are still inducer-dependent, it is likely that the actual latency for the occurrence of authentic neoplasm is much longer.

We extrapolate that all of our manipulations to transform animal or human cells may just coerce the cells into incessantly replicating, sustaining the cells' life, and manifesting neoplastic morphology and/or behavior without actually transforming the cells (Fig. 5). Therefore, when the coercion ends, the “transformed” cells either return back to the normal or undergo apoptosis as they are redundant 740-742. For instance, expression of the SV40 large T antigen in primary cells can confer additional 20-30 population doublings upon the cells, during which some cells are immortalized spontaneously 743. In animal models, the cells of the duress-sustaining lesions still have allegiance to the animal's body. This preserved allegiance, which bespeaks the non-autonomous nature and categorizes the lesions into hyperplasia (although showing malignant morphology), is the reason for why the cells regress via apoptosis so that the host organ or tissue does not possess redundant cells 203, 296, 303, 466-469,474. Actually, the lesions may show higher rates of apoptosis, and probably also senescent death, than their host tissue because of their hyperplastic nature, although the inducers may suppress the apoptosis and senescent death as components of their coercive mechanisms. In cell culture, because mortal cells in dishes are no longer under the control of the animal's body and thus do not need to care about the cell redundancy issue, the cells die of only senescent death, and not of apoptosis 302, 468. For this reason, the manipulations in most *in vitro* transformation assays are made in a perpetual manner, such as being made as stably-expressing cell clones, to prevent the loss of the coercer. Actually, some techniques of conditional immortalization and/or transformation 744-749, along with many conditionally immortalized cell lines 743, 750-759, like the temperature-controlled ones 756, 760, have been widely established to make it feasible to turn on or off the coercer gene. There are even transgenic animals established to facilitate the establishment of such conditionally immortalized cell lines 755. Some of these conditionally immortalized cells can form colonies in soft agar when the coercer is turned on, but no colony is formed when it is turned off 756, 759, 761. Obviously, the “conditional” means reversable, implying that the immortality or transformation occurs simply under the duress of the immortalizer or the transforming gene, and not due to the relevant epigenetic or genetic alterations.

In all *in vitro* and *in vivo* models, the epigenetic or genetic alteration(s) for immortality and autonomy occur only spontaneously in a random and stochastic manner during constant cell replication caused by the duress (Fig. 5). Because of this manner, it occurs only to several cells in animals at an early-enough time point that leaves the cells with a sufficient time to evolve to overt tumors. This late establishment of immortality and autonomy betokens that primary cells put more guards on the genes responsible for immortality and autonomy as the second defensive line, compared to the guards on the genes relevant to the initiation as the first defensive line, meaning that the first hit is easy, but the second hit is difficult. Until now, no exogenous agent, a chemical, irradiation, biological factor, or any other, has been identified that can break through this second defensive line of cells, and we have no idea on which genes are involved. Actually, a question that tantalizes us is whether such genes really exist. What a lengthy promotive period in all animal models established so far tells us is that breaking through this second defensive line can only be made by currently-unknown intrinsic factor(s). Fortunately, our manipulations as extrinsic factors can accelerate the breaking-through by sustaining the cells' life; accelerating cell replication, damaging DNA, and/or inhibiting DNA repair.

Regardless of its mechanism, the reversibility of *in vitro* transformation and the inducer-dependency of animal lesions remind us that we cannot consider cells or lesions neoplastic based solely on their morphology and behavior. Diagnosis of outgrowths induced in animals should not solely rest on the pathological morphology and formidable behavior, although the relentless proliferation begot by the duress will one day lead to the epigenetic or genetic alterations for true neoplastic transformation. This “coercion hypothesis” (Fig. 5), proposed by us a few years ago 720 and recently 47, 303, 466 on the essence of animal models of carcinogenesis, deserves experimental testing.

### We still lack a good strategy to determine molecular pathways leading normal cells to cancers

The genes mediating the two or three genetic hits described above remain unknown. One of the reasons is that we have been encountering a logical plight for decades regarding our research strategies and approaches, as repeatedly pointed out by us before 47, 296, 720, 762: the results from the approaches we used, such as genetic engineering, can only tell us that certain manipulations or alterations, like concomitant overexpression of the c-myc and a k-ras mutant, and the ensuing cascades of molecular changes, can eventually cause neoplastic transformation or tumor formation. However, we still do not know whether cells in humans or in untreated animals really do spontaneously develop to neoplasms because of the abnormalities of these genes and via these cascades of molecular changes. In an analogy, we have built the highway Interstate-95 (I-95) and know that Mr. Trump can go from New York City (NYC) to Washington DC by taking it, but we do not actually know whether this is indeed the path, but not one of the others, he took. If we still cannot find a way to break this impasse, our attempt to learn why and how some cells in humans become neoplastic will continue to be prodigal financially and in effort. This is because we will continue to identify (more correctly, to create) many more molecular pathways leading normal cells to neoplasms, besides the many pathways already known or created 763, while we remain unable to hold any particular pathway(s) accountable for sporadic carcinogenesis in humans. Restated, we are creating, but not identifying or discovering, pathways, such as by creating otherwise non-existing transgenic or knockout mice, and surmise that these man-made paths are the carcinogenic procedures occurring in patients' bodies. In another analogy, we already have many paths leading from NYC to DC but will endlessly build many more while remaining unable to know which path(s) Mr. Trump took or will take. Probably, we have been upending things or putting the cart before the horse in our research.

### Neglecting immortality causes confusion on aging-caused cell death in outgrowths

Normal animal cells undergo aging and eventually die of it 465, 474, 764-774, and so do cells in overt outgrowths from many animal models, more often in morphologically-benign than in morphologically-malignant ones 769, 771-773. This type of cell death has established “cancer cell senescence” as a popular research bailiwick 474, although many relevant studies do not involve lesions from animals but, peculiarly, use cancer cell lines that are immortal. Because its essence is “dies from aging”, the “cancer cell senescence” concept is illogical and collides with the immortality and autonomy criteria for a neoplastic state, since it connotes that immortal cells still undergo aging and eventually die of it. In our logic, neoplastic cells are immortal and thus cannot undergo aging and eventually die from it, whereas cells that can undergo aging and die from it cannot be regarded as neoplastic no matter how much their morphology and behavior resemble those of neoplastic cells. In all those lesions that are morphologically malignant and even metastatic but are still inducer-dependent, such as the TSH-dependent thyroid tumors and their metastases 155, 174, 185, cells may undergo aging and die via senescent death because they have not yet become authentically neoplastic.

### Neglecting immortality causes confusion on cancer stem cells

As Sell has pointed out before 4, in the mid-1990s there were two important concepts on stem cell theory of cancer reemerging simultaneously in the literature. One is the hypothesis that cancers arise from normal stem cells in the organ or tissue, which somehow had gone awry and lost the ability to differentiate while having acquired the ability to proliferate indefinitely 635. This theory actually appeared in the 19^th^ century 775 and reemerged in the literature in the 1960s and 1970s 4, 499, 632-636, 776, 777. It developed from the concept that cancers originated from embryonic cells with stemness. As reviewed by Triolo 5, this concept was first suggested by Virchow's teacher Johannes Müller in 1838, involved the work of Boll, Cohnheim, Durante, and many others, and had become popular as the “blastema theory” in the 19^th^ century. As reviewed by Trosko et al 778-783, this initial concept annotates cancer stem cells (CSCs) as those organ- or tissue-specific stem cells that somehow go amiss, likely due to some epigenetic and/or genetic aberrations 781,784, and gradually evolve to cancers 4, 634-636, 764, 785-787. Sometimes these abnormal stem cells are also called “transformed stem cells 788”, “cancer progenitor cells 789”, or “cancer-initiating cells 782, 790”. According to a denomination of carcinogenic mechanisms, in a renewable cell type a stem cell that has gone awry may stop differentiation, in Sell's words, “showing maturation arrest” 635, during an embryonic stage or during a tissue regenerative procedure, and continues proliferating to form a neoplasm, as we described before 449, 720. Actually, this “stop-differentiation” mechanism is presumably a reason why nonrenewable cell types still develop childhood neoplasms: pediatric tumorigenesis, incurred by such reasons as carriage of certain germ-line mutations or in utero exposure to a carcinogen 791, had already incepted during an embryonic stage when the cells still had their replicative ability 449. For this reason, we have suggested that molecular biologists should be wary of using those DNA elements that are activated during an embryonic stage 199, 205, such as the Mist-1 promoter 792, 793, as the promoters to drive transgenes. This is because the resulting transgenic animal models may show stop of differentiation and thus mimic only the formation of childhood cancer, whereas most cancers in humans are sporadic 199, 205. For example, both female and male transgenic mice expressing the MMTV-PyV middle T antigen develop mammary tumors at a very young age 794, in contrast to humans.

The other concept is that cancer is maintained by a small fraction of the cancer cells in the tumor mass that have the property of stem cells 795-797. In Clarke's words, “…a subset of cancer cells within some tumors, the so-called cancer stem cells, may drive the growth and metastasis of these tumors” 798. More detailed by Chiodi, “in many types of cancers a subset of cells shows peculiar characteristics, such as the ability to induce tumors when engrafted into host animals, self-renew and being immortal, and give rise to a differentiated progeny. These cells have been defined as CSCs or tumor initiating cells 799”. Similarly, in the words of Weinberg's group, “the CSC hypothesis posits the existence of subpopulations of neoplastic cells within a tumor that exhibit an elevated ability to seed new tumors upon experimental implantation in appropriate animal hosts” 800. They also say that, “evidence is accumulating that both normal and fully neoplastic cell populations harbor subpopulations of stem cells (SC) that can both self-renew and spawn more differentiated progeny” 801. This CSC definition, which is used in most recent CSC publications 518, 785, 802-806, was derived from some findings in the 1990s that many leukemia cells showed different degrees of differentiation, and a small subset of them have stem cell properties with a great potency to populate to a tumor mass when transplanted into animals 795-798, 807. Actually, many much-earlier studies, started by Furth and Kahn in 1937 808, have already shown that single cancer cells in late progression stages were highly transplantable and could grow rapidly in recipient animals 809-813, as reviewed by George Klein six decades ago 408.

The two CSC concepts described above, one about cancer-origin and the other about cancer-maintenance mechanism, are unrelated and are both correct and clear, as pointed out by Visvader 814. However, the number of the studies on the second concept has been soaring in the past 20 years, which unfortunately sets this concept as the orthodox CSC definition, admixes the two unrelated concepts together, and makes many researchers confused. For instance, CSCs are described as “cancer-initiated cells” in both concepts 782, 790, 799. Indeed, the CSC definition in the literature of the past 20 years has remained erratic or, in the words of Dumont et al 815, 816, “fuzzy and evolving”, partly because it has never been lucid in distinguishing CSCs on the one hand from normal stem cells in embryos or in adult organs and on the other hand from the vast majority of cancer cells 817.

The “subset”, “subpopulation”, or similar words used in the second CSC concept about a cancer maintenance mechanism hint slyly, in a tacit manner, that except for a tiny fraction, the vast majority of cancer cells are not immortal and are not able to self-renew, which obviously collides with the definition of neoplasm in all pathology textbooks published since the 1900s. Benign and malignant cells relentlessly undergo symmetrical binary fission, just like bacterial cells that unremittingly divide to maintain their strains, although some aging research wizards consider that bacterial cells also undergo asymmetrical division and undergo aging as well 818-820, likely for maintaining their vitality 821. Moreover, malignant cells are highly plastic and can differentiate to various cell types 807,822. For instance, quite different types of cancer, even pre-cancer lesions 823, of the epithelial origin 824-834 manifest bone histology, or osseous metaplasia in pathological phraseology. Therefore, a strong pluripotency should not be used to dichotomize cancer cells into CSC and non-CSC groups. It is true that in cancers many cells die at a much higher rate than others due to various stressors, such as insufficient oxygen or nutrient nourishment, or overly severe genetic damage 554, 555, 835. The opposite is also true that in a cancer mass some cells' ability of self-renewal via symmetrical division is much more potent than that of the others. However, these quantitative differences simply reflect the well-known heterogeneity of malignant cells 453, 836-840, which is largely ascribed to the stemness of some cells 841 and the great genetic variation among most cells 835, 842, and should not be used to split cancer cells into CSC and non-CSC groups either. More critically, “ability of self-renewal *vs* inability of self-renewal” is actually “immortality *vs* mortality”, which is a black-and-white demarcation between the neoplastic and non-neoplastic states and thus should not be used again as the demarcation between CSCs and non-CSCs. We would like to quote Paget's words in 1889 again: “as Langenbeck says, every single cancer cell must be regarded as an organism, alive and capable of development” 304; obviously, both Paget and Langenbeck used “every single” to extend the “self-renewal” ability to all cancer cells.

We proffer that, since CSCs in the second definition differ from other cancer cells only quantitatively in such as the competency of self-renewal, metastasis, therapy-resistance, etc., clear quantitative parameters in these vicious behaviors should be established to separate those highly-competent cells from their less-competent counterparts, just like the establishment of the normal ranges for blood pressure, blood sugar, etc. Identification of biomarkers for these cells, as performed by many cancer researchers now, is part of this line of work. Once these quantitative parameters have been set as criteria, these abominable cells can be more easily defined, identified and studied for their behaviors in the quarters of chemotherapy, metastasis, patients' prognosis, etc., without calling them CSCs. For example, it is unnecessary to call “CD44(+)/CD24(-) and ALDH1(+)” breast cancer cells as CSC 843-846, since “CD44(+)/CD24(-) and ALDH1(+)” defines them more specifically and clearly than the equivocal “CSC”. Meanwhile, returning the CSC concept to the cancer-origin hypothesis will be helpful to solve the current snafu in this realm of cancer research.

Mention should be made of some publications which prefer not to provide a pellucid CSC definition, like the ones by Sarkar 847-849, but, instead, prefer to sway between different definitions. In this type of even more baffling definitions, a CSC could be a normal stem cell that eventually evolves to a cancerous one, the benign stage towards a cancerous cell, or the earliest form of a cancerous cell. While some peers, such as Reya et al 850,851, seem to consider both cancer-origin and cancer-maintenance types of cells as CSCs, some others, such as Chaffer and Weinberg 801, sometimes consider that those cancer-maintaining CSCs, but not all cancer cells of the cancer mass, may be derived from tissue-specific normal stem cells in a stepwise manner. These latter cancer pundits elegantly incorporate the hypothesis on cancer-maintenance mechanism into the notion of normal-stem-cell origin of cancer, but it still indirectly suggests that only CSCs, and not the vast majority of cancer cells in the tumor mass, can self-renew, and thus still denies immortality as a *sine qua non* for a neoplastic state.

In our long-term cogitation on the hypothesis of CSC as “a”, if not “the”, mechanism for cancer maintenance, an intriguing question occurs to us that whether benign tumors also utilize their “benign tumor stem cells (BTSC)” for their maintenance. As implicated in some of the above sections, benign tumor cells are also immortal and thus have acquired self-renewal ability, although they may be too differentiated to be distinguishable from their normal counterparts in cellular morphology, with uterine leiomyoma as an epitome. Is the continuing expansion of a benign tumor mass also ascribable to a tiny subset of BTSCs, but not to the vast majority of the tumor cells? Otherwise, why does a cancer have to be maintained by a tiny subset of cells?

## Concluding remarks

Over a century ago, pathologists had set immortality and autonomy as indispensable canons for neoplasms, including the benign ones. It has been known ever since the 1900s that many overgrowths induced in animals are inducer-dependent until very late stages. Unfortunately, indubitable evidence for immortality and autonomy has not been provided for the lesions resulting from most genetically modified animal models of carcinogenesis at the time when the lesions are collected from the animals. Although these lesions have indeed provided us with a profusion of information on the functions and underlying mechanisms of the manipulated genes, especially on the quarters of cell proliferation and death, the lack of this needed evidence still makes us apprehensive, as much confusion on the behaviors of these animal lesions may be caused by their neoplastic inauthenticity. There are five lines of phenomena that dissent from the orthodox doctrine of cancer theory but should not be neglected, i.e. 1) the extrication of one from another among transformation, immortality, and autonomy; 2) the reversibility of *in vitro* transformation; 3) the irrelevance of mutation to cancer formation and cancer cell heterogeneity in some experimental systems; 4) the inducer-dependency of outgrowths induced in animals; and 5) the insinuation of a poor *in vivo* transformation efficacy by a low tumor yield in each animal of most model systems after a long latency. Probably, our *in vitro* and *in vivo* manipulations cannot mimic most situations of human cancer formation in which mutations are likely required for early establishment of immortality and autonomy. We should, with numerous genetic models created, embark on the following research quests: 1) What is the genomic basis for immortality or autonomy? 2) What is the genomic basis for initiation, promotion, or progression, or for the first, second, or third genetic hit outlined herein? 3) How are immortality and autonomy linked to initiation, promotion, and progression? 4) Which are good animal models for us to use in tackling the above three tasks? Concerns raised in this essay are obviously provocative but deserve reconsideration by cancer researchers, especially those at the pinnacle of cancer research.

## Figures and Tables

**Table 1:** Five common types of tumor tissue transplantation.

Name	Definition
Autologous	transfer back elsewhere in the same animal
Homologous I	transfer to a tumor-bearing animal of the same species
Homologous II	transfer to a normal animal of the same species
Heterologous I	transfer to a tumor-bearing animal of a different species
Heterologous II	transfer to a normal animal of a different species

## Abbreviations

APC: adenomatous polyposis coli
ACI: August strain crossed with Copenhagen strain
CSC: cancer stem cell
DC: Washington DC
DMBA: 7,12-diemthylbenz(a)anthracene
HTLV-1: human T cell lymphotropic virus type I
HP: helicobacter pylori
MMTV: mouse mammary tumor virus long terminal repeat
RB: retinoblastoma protein
NYC: New York City
SASP: senescence-associated secretory phenotype
SD: senescent death
TSH: thyroid stimulating hormone
